# Journal De La Physiologie De L’homme Et Des Animaux, Publié Sous La Direction Du Docteur E. Brown Sequard

**Published:** 1858-05

**Authors:** 


*Journal de la Physiologie de l'homme et des animaux, Publié sous la direction Du Docteur E. Brown-Séquard.*

We have received the first number of this Journal, which our readers will recollect as announced by us a month or two since. Its arrival we hail with satisfaction, from the benefit and pleasure which we expect personally to derive from its pages; and we are confident that the ends which this journal proposes to accomplish, in furnishing an exclusive medium for researches in the department of Physiology and the kindred branches of science, will be amply fulfilled.

It is the purpose of the editors to present all the principal original labors of the French physiologists, and an historical *resumé* of the progress and changes of physiology in all countries. "Thanks to their personal relations with most physiologists of our day in Europe and America, the editors of this journal, among whom the editor in chief is happy to name, Dr. Ch. Robin, Ch. Ronget and Tholozan, will readily procure all the important publications (journals, books, theses, pamphlets, etc.) which have for their object human and comparative physiology, and will thus give, as complete a *resumé* as possible of the progress of this science.

"We are happy to announce that we are able to count upon the collaboration of most of the French physiologists, and many physiologists of Germany, England, and America, and we believe in consequence, that we may seriously promise that all the numbers of this journal will be rich in original memoirs, not solely from the ordinary editors, but from a large number of other physiologists.

"The authors who may wish to publish their articles in this journal, will here have an absolute freedom of scientific discussion; but the editor in chief deems it his duty to state in advance, that the object of this publication is purely the advancement of science, and that personal polemics will invariably and completely be excluded."

"This journal will have for its object, in addition to pure physiology,

1*st.* *Organic chemistry, hygiene, toxicology and legal medicine, in their relations with physiology.*

2*d.* *Descriptive anatomy, comparative anatomy, teratology, and normal and pathological histology, in as far as they throw light upon physiolo*[illegible]

3*d.* *The application of physiology to the practice of medicine, surg*[illegible] *and obstetrics.*"

We have translated enough of the prospectus to give our readers an idea of the objects which this journal has in view; its first number is issued in admirable style by Bailliere and Son, of Paris, and contains 216 pages, making a volume of about the size of the North American Medico-Chirurgical Review. It will be published quarterly, and can be subscribed for by application to B. Waterman & Co., 290 Broadway, New York, or J. Pennington, of Philadelphia. The price of subscription in this country, is 25 francs, or about $5.

In this number we notice articles from Brown-Séquard, Robin, Blendiot, and other eminent French physiologists, as well as Bence Jones, and Dickinson, of England, and F. G. Smith, of our own country.

We are, indeed, gratified to be able to anticipate that some of the labors of our countrymen will at last become known to the French before the lapse of a half a century; and though our facilities for the study of these branches are not so great, and though our government is insensible as yet to the importance of scientific advance, yet we have produced, and will produce works with which it is incumbent upon scientific men of all countries to become acquainted, and we hope that our eminent physiologists will make use of this avenue, as freely as the importance of the subject demands.

